# Ethnoveterinary Study of Plant-Based Remedies for Treating Diseases in Small Ruminants in Maputo Province, Mozambique

**DOI:** 10.1155/2023/1842870

**Published:** 2023-10-05

**Authors:** Filomena M. A. Barbosa, Aida C. Cala, Viktor Sevastyanov, Ernesto Boane, Delfina F. Hlashwayo

**Affiliations:** ^1^Department of Biological Sciences, Faculty of Sciences, Eduardo Mondlane University, Maputo 1101, Mozambique; ^2^Directorate of Animal Sciences, Institute of Agrarian Research of Mozambique, Maputo 1109, Mozambique; ^3^Department of Chemistry, Faculty of Sciences, Eduardo Mondlane University, Maputo 1101, Mozambique

## Abstract

Small ruminants, particularly goats and sheep, are key livestock species in Mozambique, and their production is mainly undertaken by families. However, small ruminants are often plagued by diseases that can cause considerable economic damage. In this context, traditional remedies, including various plant species, have been widely used to manage these diseases. The aim of this study was to explore the use of plant-based remedies and other treatments for managing diseases in small ruminants in Maputo Province, Mozambique. Data collection involved conducting interviews with 44 small ruminant breeders across 5 districts in Maputo Province to identify the plants and other remedies commonly used for managing diseases in their animals. We identified a total of 38 plant species belonging to 22 families. Among these plants, four were not identified by their scientific name. The most important plants reported were *Cissus quadrangularis*, *Euphorbia kirkii*, and *Aloe* sp., with *Cissus quadrangularis* being particularly noteworthy as it was frequently cited for the treatment of wounds. The most commonly cited botanical families were Fabaceae, Euphorbiaceae, and Asphodelaceae. Interestingly, in addition to plant-based remedies, we also identified other nonplant sources of treatment, such as alkaline batteries, which are commonly used to treat wounds in small ruminants. The use of plants for ethnoveterinary purposes remains prevalent in Maputo province, with older breeders serving as the primary custodians of this traditional knowledge. Efforts should be made to document and share the knowledge of these older breeders, ensuring that it is not lost over time. This preservation of ethnoveterinary knowledge can contribute to sustainable livestock management and support the wellbeing of both rural communities and their animals.

## 1. Introduction

Mozambique is a developing country with vast livestock potential and extensive flora where the use of medicinal plants is a viable and cost-effective alternative for animal welfare. This is particularly important in rural areas, where access to veterinary services is often restricted and expensive. The livestock sector plays a significant role in the country's agrarian economy, contributing to economic development and serving as a source of family income and social and cultural prestige. In addition, livestock plays an important role in increasing agricultural production through animal traction and the use of manure [1].

Small ruminant production, particularly of goats and sheep, is a dominant livestock activity in Mozambique, which is largely driven by the family sector. These animals are often raised in natural pastures, requiring minimal investments in housing, healthcare, and food [2].

Goats are the most commonly reared livestock species in Mozambique, surpassing sheep. However, the full potential of small ruminant production has not yet been realized, as these animals are susceptible to various diseases. These diseases pose significant challenges to small ruminant production in Mozambique. Besides gastrointestinal illnesses, which are a common concern causing economic losses due to reduced growth and weight loss, there are other health issues affecting these animals. The tropical climate in Mozambique provides an environment conducive to the spread of infectious agents [3]. In addition, the sharing of pasture areas and water sources with wildlife further increases the risk of disease transmission. Overcoming these disease challenges is crucial for maximizing the potential of small ruminant production and ensuring the overall wellbeing and profitability of livestock farmers in Mozambique.

In rural areas of Mozambique, households often lack the financial resources to purchase veterinary drugs, which has led to a search for alternative treatments using locally available resources that can provide comparable or superior results to conventional products. Many rural communities rely on medicinal plants and other traditional remedies to manage the health of their livestock in the country.

Despite the potential benefits, there is a lack of extensive research on the use of these alternative methods for veterinary practice in Mozambique and there is often a lack of scientific validation of these practices [4]. Ethnoveterinary studies can help to bridge this gap by documenting traditional knowledge and practices, evaluating their effectiveness, and identifying potential risks and benefits. Such studies can provide valuable information for animal health professionals and policymakers, as well as for local communities who rely on traditional knowledge and practices for the management of their livestock.

The purpose of this study was to identify the plants used to treat diseases in small ruminants in Maputo. We gathered information on the specific parts of the plants used, the methods of preparation, the administration routes, and any reported adverse effects. In addition, we collected data on nonplant-derived products used for treating animals. Thus, this study provides valuable insights into the use of local resources for veterinary care.

## 2. Materials and Methods

### 2.1. Study Area

The study was conducted in rural settlements across five of the eight districts in Maputo Province. The selected districts were Boane, Marracuene, Moamba, Matutuine, and Namaacha, chosen for their known presence of small ruminant breeders and easy accessibility.

Maputo Province, the southernmost province of Mozambique, has an area of 22,693 km^2^ and a population of 1,968,906 inhabitants according to the latest census in 2017 [5]. The province's capital city is Matola. It shares borders with Gaza Province to the north, the Indian Ocean to the east, the South African province of KwaZulu-Natal to the south, Eswatini to the southwest, and the Mpumalanga province of South Africa to the West and Northwest.

### 2.2. Ethical Compliance

This study adhered to the ethical standards outlined by the International Society of Ethnobiology (ISE) Code of Ethics [6], as well as the local legislation on traditional knowledge [7]. The head of the department of biological sciences at Eduardo Mondlane University provided authorization for the project after obtaining approval from the scientific committee. In addition, authorization to conduct research was obtained from each District Office of Economic Activities (*Serviço Distrital de Actividades Económica*s (SDAE)). Before data collection, veterinary doctors and technicians from SDAEs accompanied the investigators to meet with the community's elders, known as “*régulos*”, and small ruminant breeders to obtain their permission to participate in the study. Before participation, the study's objectives were thoroughly communicated, and all breeders who participated in the interviews provided informed consent verbally.

### 2.3. Collection of Ethnoveterinary Data

From December 2018 to February 2019, ethnoveterinary data were collected during 10 field visits across five districts. Each district was visited twice. Snowball sampling was employed, and interviews were conducted in the local language (*Xitsonga/Xichangana*) or Portuguese, depending on the informant's preference. Following the initial interview, we requested that the first informant refer us to other small ruminant breeders who had knowledge regarding medicinal plants and other remedies used to treat goats and sheep. In total, we conducted interviews with 44 small ruminant breeders. Semistructured forms were used to capture the interviewee's personal information and data regarding the ethnoveterinary use of plants and other remedies. We also collected data on the most common diseases affecting the animals, the plants, and other remedies used for the treatment, and the specific diseases treated, preparation methods, administration routes, potential side effects of the plants, and other treatments used.

### 2.4. Plant Collection and Identification

During the data collection process, voucher specimens of the medicinal plants mentioned by the informants were collected from local forests or the homes of the breeders. These specimens were deposited at the Eduardo Mondlane University's Herbarium (LMU) in Maputo, Mozambique.

To ensure the accuracy of the identification process, botanist technicians from the university were responsible for identifying the collected plant specimens. The plant names were cross-checked with the World Flora Online [8]. Botanical families were classified according to the Angiosperm Phylogeny Group IV system (APG IV) [9]. We conducted research on the IUCN status of each plant mentioned, utilizing https://www.iucnredlist.org/. In addition, we explored the recent literature concerning the vascular plants of Mozambique [10] to determine the plant's endemism within Mozambique and its classification as native or introduced, using the same reference.

### 2.5. Data Analysis

To conduct a quantitative analysis of the ethnobotanical data, we utilized three different calculations: relative frequency of citation (RFC), use value (UV), and fidelity level (FL). RFC was calculated by dividing the frequency of citation of a particular species (FC) by the total number of interviewees (N). The formula used was RFC = FC/N [11]. UV was calculated by dividing the number of uses mentioned by each informant for a specific plant species (Ui) by the total number of interviewees (N) (UV = Ui/N) [12]. Finally, FL was calculated by dividing the number of informants who reported a specific use of a plant species (Np) by the total number of informants who mentioned the plant for any purpose (Ns) (FL = Np/Ns) [13].

The assessment of the association between sociodemographic factors and the utilization of plants for treating small ruminants was conducted using Epi Info™ version 7.2.5.0. The chi-square test and Fisher's exact test were employed due to their ability to examine relationships between categorical variables. The sociodemographic factors under investigation encompassed age group, gender, place of birth, profession, religion, residency, schooling, and years of experience in small ruminant breeding. Associations were considered statistically significant if the *P* value was less than 0.05.

## 3. Results

### 3.1. Demographic Characteristics of the Study Respondents

In this study, a total of 44 informants were included, with only 7 (15.9%) of them being female. The average age of the informants was 57 years, although 3 of them did not remember their age. The age of the informants did not exhibit a statistically normal distribution. All the informants were born in Mozambique, with the majority (77.3%, *n* = 34) being from the Maputo Province. Some informants were originally from other provinces where they acquired traditional knowledge.

In terms of religion, 77.3% (*n* = 34) of the informants identified as Christians from diverse denominations. The majority of the informants worked exclusively in small ruminant breeding (61.4%, *n* = 27), while the remaining participants had other professions (38.6%, *n* = 17) such as tailor, typist, entrepreneur/trader, veterinary technician, house security guard, retired, mechanical, police, manufacturer of wooden stakes, bricklayer, traditional healer, secretary on the district executive council, charcoal maker, and store attendant. None of the informants had a university degree, and most of them completed primary school (1^st^ to 5^th^ grade) under the former education system (36.4%, *n* = 16). Around a third of the informants (34.1%, *n* = 15) did not attend school. Many of the informants had over 30 years of experience in small ruminant farming. A summary of the demographic characteristics of the informants is presented in Table 1.

### 3.2. Use of Plants and the Source of Traditional Knowledge

Out of the total 44 informants in the study, 14 (31.8%) did not use plants to treat their animals. Among the remaining 30 (68.2%) who reported using plants, only 6 did not use them in the 12-month period preceding the interview. The majority of the informants (61.4%, *n* = 27) used plants in conjunction with veterinary medicines, while 3 informants (6.8%) used plants in combination with chemical products. Interestingly, none of the informants reported observing any side effects of the plants they used.

**Figure 1:**
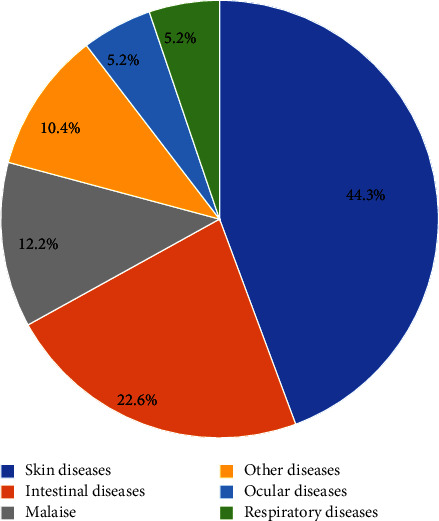
Diseases that mostly affected the animals in the study areas.

The informants identified skin diseases and intestinal disorders as the most common illnesses affecting their animals, as shown in Figure 1.

The study found that the most common way for the informants to learn about plants used medicinally was from their parents and relatives, accounting for 34.1% (*n* = 15) of the respondents. Fourteen informants (31.8%) did not provide a response to this question. Other ways of learning included from other small ruminant breeders (15.9%, *n* = 7), grandfathers (9.1%, *n* = 4), self-learning (4.5%, *n* = 2), and work colleagues (4.5%, *n* = 2). In terms of plant collection, most of the informants reported collecting plants from nearby forests.

### 3.3. Plant Species and Other Remedies Used to Treat Diseases in Small Ruminants

The study identified 38 plant species used for addressing health issues in small ruminants. However, due to the unavailability of specimens, scientific names for 4 plants were not determined.  Table 2 provides a comprehensive summary of the plants and their corresponding ethnoveterinary uses for the treatment of diseases in small ruminants. Notably, *Cissus quadrangularis*, *Euphorbia kirkii,* and *Aloe* sp. were the most commonly cited medicinal plants with RFC values of 0.45, 0.20, and 0.16, respectively. In addition, other frequently mentioned plants included *Aloe marlothii*, *Dietes iridioides*, *Psydrax locuples*, *Terminalia sericea*, and *Vernonia* sp., all with an RFC of 0.07.  Table 3 provides information on the FL of the most cited plants for specific ailments.

**Figure 2:**
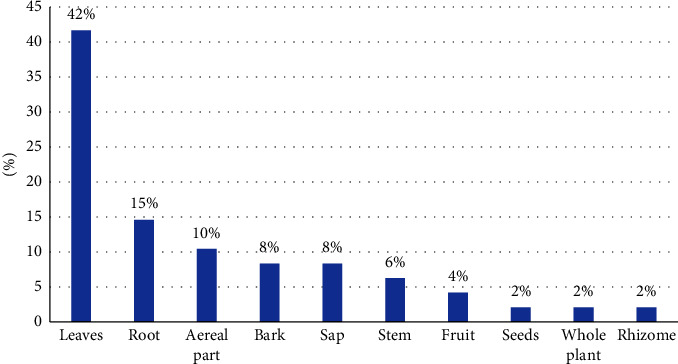
Percentage of use of plant parts.

Figure 2 illustrates that the leaves of the cited plants were the most frequently utilized part, followed by the roots and other less commonly used plant components.

**Figure 3:**
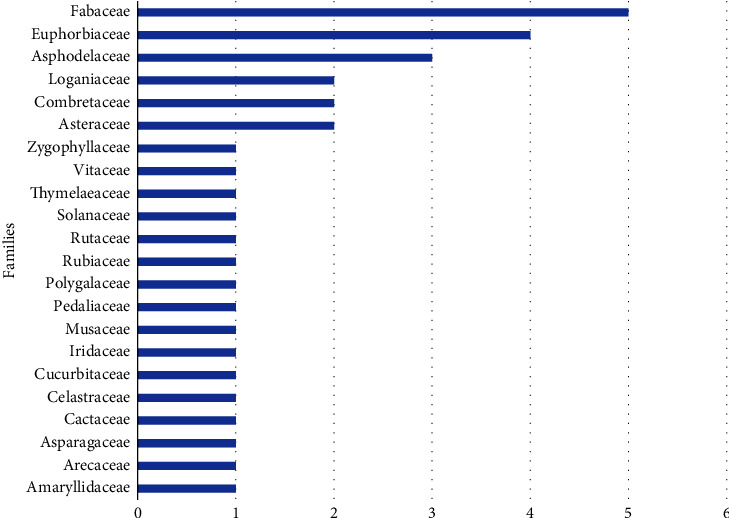
Overall distribution of plant species across different families.

As illustrated in Figure 3, the botanical families that occurred most frequently were Fabaceae, Euphorbiaceae, and Asphodelaceae.

**Figure 4:**
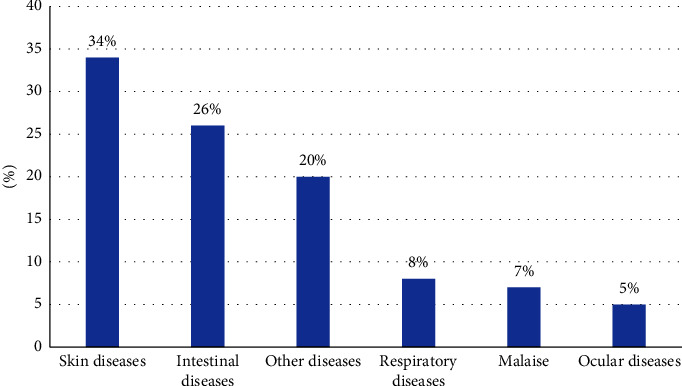
Percentage of plants used for treating different ailments in small ruminants.

As indicated in Figure 4, the majority of plants were utilized for treating skin ailments.

The districts of Namaacha and Matutuine were identified as having a higher number of mentioned plant species (14 and 12, respectively), while Boane, Moamba, and Marracuene districts had a lower number (10, 9, and 7, respectively). None of the sociodemographical factors exhibited a statistically significant association with the use of plants, as determined by both the chi-square test and Fisher's exact test (Table 4).

Regarding the IUCN status, none of the plants were found to be endangered based on the information we gathered from the IUCN Red List. In particular, out of the 27 plants that were identified at the species level, 26 were classified as “least concern,” while data for *Opuntia ficus-indica* are not available. In terms of endemism, only *Aloe marlothii* and *Alantsilodendron pilosum* were designated as “near-endemic,” based on the criteria outlined in the updated checklist of Mozambique's vascular plants [10]. Information for the remaining species was not available. The concept of “near-endemic,” as defined in the referenced article, pertains to species occurring in five or fewer localities beyond Mozambique.

Regarding the nativity or introduction status of species, within the subset of 27 plants with complete species-level names, only *Nicotiana tabacum* and *Opuntia ficus-indica* were recognized as introduced species. Conversely, all remaining species were categorized as native. Supplementary File 1 contains a table detailing the IUCN status, endemism, and nativity of the species.

In addition to plants, other remedies used for treating small ruminants were mentioned, with alkaline batteries being the most commonly cited (mentioned by 6 informants) for wound treatment (as presented in Table 5). Other nonplant remedies included lichen, internal shell of cuttlefish, water from car batteries, burned lubricant oil from car motors, petroleum, and oil, all of which were used to treat diseases in small ruminants.

## 4. Discussion

The findings of this study reveal that small ruminant breeding is still prevalent in Maputo Province, and that traditional remedies, including plants, are commonly used to treat these animals. The informants' low level of education, with most only attending school up to 5^th^ grade, may be attributed to the long distances to schools in rural areas, causing many students to abandon their education.

Typically, individuals with low levels of schooling tend to hold more traditional knowledge. Some breeders also reported having other professions, which may be attributed to urbanization in the districts. The informants in this study had significant experience in small ruminant farming, with more than half (68.2%) using plants to treat their animals. This demonstrates that knowledge of medicinal plants remains prevalent in the province, with much of it acquired through family and colleagues. However, this knowledge is at risk of erosion due to urbanization and the availability of veterinary services. As such, careful consideration of this matter is essential, given the importance of preserving the country's traditional knowledge.

The study revealed that a total of 38 plant species were cited for treating diseases in small ruminants. Among these plants, *Cissus quadrangularis* had the highest FL of 1 for the treatment of wounds. The plant has been recognized for its medicinal properties in various traditional systems of medicine of the African continent [14–19]. Its use in wound healing can be attributed to its reported anti-inflammatory, analgesic, and antimicrobial properties. The plant is known to contain bioactive compounds such as flavonoids, terpenoids, alkaloids, and other phytochemicals, which may contribute to its therapeutic effects [20–22].

The high FL score of 1 indicates that *C. quadrangularis* is exclusively used for treating wounds in small ruminants within the local community. This finding suggests a strong cultural and empirical knowledge of the plant's effectiveness in addressing this specific health condition. Further research is warranted to explore the mechanisms of action and validate the traditional use of *Cissus quadrangularis* in wound healing. Such investigations may involve studying its chemical composition, conducting preclinical studies to assess its efficacy, and eventually conducting trials to establish its safety and effectiveness in small ruminants.

The plant with the second highest FL was *Euphorbia kirkii*, scoring 0.89. According to local knowledge, applying one or two drops of the plant's sap on the skin between the eyes and ears, where the optic nerve passes, can effectively treat keratoconjunctivitis and other eye diseases in small ruminants. This practice is commonly known and shared by many breeders across different districts. Further research is necessary to identify the chemical compounds in the plant that exhibit pharmacological activity and to determine the mechanisms of action. Since keratoconjunctivitis is often caused by bacterial infections, it is possible that the plant's active compounds have antibacterial properties.

The commonly cited plant, *Aloe* sp., with an FL of 0.29, is well-known on the African continent for its use in the treatment of diarrhea and roundworms. This genus has been extensively studied and has already led to the development of several pharmaceuticals. Other ethnobotanical studies in Mozambique have also mentioned the antidiarrheal properties of *Aloe* species [4, 23]. Similarly, studies from other African countries have reported the usefulness of *Aloe* species in treating diarrhea and roundworms [24]. Some species within this genus have been studied for their phytochemical profile, such as *Aloe vera* [25]. Moreover, similar to our findings, plants from the *Aloe* genus have also been commonly used for the treatment of wounds in small ruminants in other countries [26–28]. As intestinal diseases have also been a common complaint among small ruminant breeders, it is important to continue studying this plant genus, particularly in regard to its antiparasitic and antibacterial properties.

In this study, informants commonly cited *Aloe marlothii*, *Dietes iridioides*, *Psydrax locuples*, *Terminalia sericea*, and *Vernonia* sp., all with a relative frequency of citation (RFC) of 0.07. These plants were frequently employed for managing various ailments, primarily diarrhea, and other health conditions. However, it is worth noting that *Psydrax locuples* held a unique position as it was specifically used for treating wounds and furuncles.

*Dietes iridioides*, although lacking laboratory studies, is traditionally used in South Africa for treating diarrhea and dysentery using its rhizomes [29]. Another *Dietes* species, *Dietes bicolor*, has been subjected to antimicrobial tests against bacteria and fungi, demonstrating potent antimicrobial activity [30]. Considering the botanical proximity between *Dietes iridioides* and *Dietes bicolor*, as well as the shared traditional use of *Dietes iridioides* for treating diarrhea, worms, and cough, further research is necessary to investigate the potential antidiarrheal properties of *Dietes iridioides*.

Mozambican researchers investigated the antimicrobial and analgesic activity of aqueous and ethanol extracts derived from the leaves of *Psydrax locuples*. Their research findings demonstrated the remarkable antimicrobial activity of extracts derived from the plant, surpassing the effectiveness of the recommended drugs. These extracts exhibited efficacy against a range of microorganisms, including bacteria and fungi [31]. This supports the traditional use of *Psydrax* plants in African traditional medicine, where they are extensively employed for treating various health conditions such as malaria, fever, headaches, edema, rheumatism, diarrhea, conjunctivitis, mycoses, and other minor ailments [32].

Our study revealed that breeders commonly rely on *Psydrax locuples* for the treatment of wounds and furuncles in small ruminants. This practice aligns with the wider utilization of *Psydrax* plants in African traditional medicine for diverse therapeutic purposes. Furthermore, the potent antimicrobial activity of *Psydrax locuples* supports their effectiveness in addressing dermatological conditions [31]. This antimicrobial activity is particularly valuable in combating infections that may be associated with wounds and furuncles. The ability of the plant to target a wide range of microorganisms contributes to their potential as therapeutic agents for these conditions. However, it is important to note that the efficacy of *Psydrax locuples* in treating wounds and furuncles may also involve other mechanisms beyond their antimicrobial activity. Further research can explore additional mechanisms and properties of *Psydrax* plants to enhance our understanding of their therapeutic effects on wounds and furuncles.

Our study highlighted *Terminalia sericea* as a remedy for managing abdominal pain, diarrhea, and cough. This medicinal plant is highly regarded in African traditional medicine, particularly in Southern Africa, where it holds a significant place [33, 34]. The historical use of *T. sericea* roots in relieving diarrhea has been extensively recorded [35]. In addition, numerous scientific studies have substantiated the plant's ability to combat various bacteria through its antimicrobial properties [36–38].

While the existing evidence from traditional use and scientific research is promising, further studies are needed to fully understand the therapeutic potential of *T. sericea*. Additional research could include investigations into the active compounds present in the plant and their mechanisms of action. This knowledge could pave the way for the development of standardized formulations and dosage recommendations. Moreover, it would be valuable to conduct veterinary clinical trials, to evaluate the efficacy and safety of *T. sericea* in managing the mentioned conditions. These studies would provide more robust evidence and contribute to the integration of the plant into evidence-based veterinary practices.

Another commonly mentioned species in our study, *Vernonia* sp., has a wide range of medicinal uses, including wound treatment. Previous studies have demonstrated their antiulcer properties [39], as well as their potential as antibiotics and antibiofilm agents [40], yielding promising results. Recent research focused on the methanolic extract of *Vernonia elaeagnifolia* aerial parts, which showed a significant decrease in ulcer index in rats, comparable to the standard drug omeprazole. In addition, *Vernonia condensata* Baker has been traditionally used for the treatment of inflammatory and infectious conditions. Studies have highlighted the leaves of this plant as a promising natural source of active compounds effective against various pathogens, including multidrug-resistant strains [40].

Overall, the findings regarding the IUCN status of the studied plant species are quite encouraging, as none of them were identified as endangered according to the IUCN Red List. Out of the 27 plants with species-level identification, a vast majority, 26 to be exact, were classified as “least concern,” indicating that they currently do not face significant threats to their survival.

The analysis of endemism revealed that *Aloe marlothii* and *Alantsilodendron pilosum* were classified as “near-endemic,” implying that they have a relatively restricted distribution. These species may require special conservation attention, as their localized occurrence makes them potentially more vulnerable to environmental changes and threats within their range.

Considering the nativity or introduction status, the fact that nearly all identified species were native raises an interesting point. It strongly suggests that the local population's dependence on native plants for animal treatment may be a practice deeply rooted in ancient knowledge, dating back to a time before the introduction of other, nonnative plants. This underscores the longstanding tradition and extensive understanding of the plants' therapeutic qualities within the community.

In regard to the utilization of alternative nonplant-based remedies, the use of alkaline batteries as a remedy for treating small ruminants is a cause for concern, as it was reported by many informants besides medicinal plants. This raises the question of whether there are any chemical compounds in the batteries that could potentially have a positive effect on wound healing or antibacterial activity. Further research is needed to investigate this possibility, as well as to assess the potential toxicity of using alkaline batteries for medicinal purposes. It is also important to educate and inform communities about the potential risks associated with using such products for medical purposes, to prevent any harm to animals.

The use of chemical products in animal treatment may be attributed to the increase in commercial activities in rural areas, leading breeders to experiment with different remedies. However, it is important to carefully analyze the use of such products, as they may contain high concentrations of toxic compounds that can have adverse effects on the animals.

Interestingly, the internal shell of cuttlefish has also been reported in another ethnoveterinary study conducted in Italy for the treatment of eye infections and diseases, similar to what was mentioned in this study [41]. This highlights the potential for natural and unconventional sources of remedies that can be explored for their efficacy and safety in veterinary medicine. These findings also suggest that the use of natural products for animal treatment may be a common practice across different regions and cultures.

Livestock breeding in Maputo Province is still largely supported by natural pastures, however, the province is undergoing rapid urban development driven by housing, industrial, and commercial construction. This reduction in biodiversity is reducing the use of medicinal plants and wild forage by local populations while simultaneously increasing access to veterinary services. These findings underscore the importance of continuing to develop studies aimed at recording traditional knowledge for future use.

The less urbanized and more remote districts, such as Namaacha and Matutuíne, recorded a higher number of medicinal plants compared to the more urbanized districts (Boane, Moamba, and Marracuene). This suggests that the lack of conventional veterinary services in these areas may be driving the reliance on traditional remedies.

This study highlights the urgent need for the preservation of local knowledge as there is a great risk of losing valuable information, especially since most of the knowledge holders are of old age.

To ensure the ethical and equitable sharing of knowledge, in accordance with the principles of access and benefit sharing, we are committed to return the findings of our ethnoveterinary study to the holders of traditional knowledge who generously contributed their insights. We will share the acquired knowledge and also provide insights into scientific research conducted on the plants, as well as their endemic status.

In addition to these community-based efforts, we recognize the significance of extending our awareness-raising initiatives to a broader scale. To achieve this, we plan to build upon the existing work conducted within the university, which involves engaging with various media platforms such as television and newspapers. Through these channels, we will emphasize the importance of preserving traditional knowledge and specifically focus on the traditional plant knowledge within the veterinary context. We intend to highlight the ongoing studies conducted by the university and the knowledge generated from these endeavors.

Furthermore, we aim to collaborate closely with local organizations to expand the reach of our research findings to other communities across the country and raise awareness about the significance of this knowledge. We also aim to emphasize the importance of exercising caution when using chemical products as many of these products have adverse effects on animal health.

Moreover, we are committed to participating in traditional medicine symposiums held in the country. During these events, we will bring this topic to the forefront of discussions and continue to advocate for the establishment of a comprehensive and unified plan for ethnobotanical studies in Mozambique. Such a plan would facilitate the documentation of traditional knowledge, benefiting both knowledge holders and the scientific community. By combining these initiatives, we aim to create a synergy that not only respects and preserves traditional knowledge but also actively contributes to its recognition, appreciation, and application within the context of veterinary and broader traditional medicine practices in Mozambique.

## 5. Conclusions

In this study, we documented the use of 38 plant species belonging to 22 families for the treatment of diseases in small ruminants. Among the most important plants were *Cissus quadrangularis*, *Euphorbia kirkii*, and *Aloe* sp., with *Cissus quadrangularis* frequently cited for wound treatment. Notably, remedies of nonplant origin, such as alkaline batteries, were commonly used for wound treatment in animals. The study highlights the ongoing utilization of traditional plant-based treatments for animal diseases in Maputo Province, underscoring the need to preserve and register this knowledge.

Furthermore, this study underscores the importance of conducting research on the biological activity of plants belonging to the national flora. This work is currently underway at the University and may lead to the scientific validation of traditional medicinal plants, as well as the development of low-cost and accessible products for local livestock farmers who lack access to veterinary services. In addition, the study draws attention to the potential adverse effects of chemicals used by breeders on animal health, calling for further research to document both plant-based and nonplant-derived remedies for animal diseases.

In summary, this study contributes to the growing body of research on traditional plant-based treatments for animal diseases and highlights the need for continued scientific investigation to validate the scientific basis for such traditional practices and to preserve the traditional knowledge.

## Figures and Tables

**Table 1:** Demographic characteristics of study respondents.

Variables	*N* (%)
*Gender*	
Male	37 (84.1%)
Female	7 (15.9%)
*Age range*	
25–39	4 (9.1%)
40–59	18 (40.9%)
60–79	16 (36.4%)
80–89	3 (6.8%)
Did not remember age	3 (6.8%)
*Place of birth*	
Maputo	34 (77.3%)
Inhambane	5 (11.4%)
Gaza	1 (2.3%)
Nampula	1 (2.3%)
Not reported	3 (6.8%)
*Religion*	
Christian	34 (77.3%)
Traditional African religion	4 (9.1%)
Muslim	3 (6.8%)
No religion	3 (6.8%)
*Schooling*	
1^st^–5^th^ grade (former system)	16 (36.4%)
1^st^–12^th^ grade (actual system)	12 (27.3%)
No schooling	15 (34.1%)
Literacy	1 (2.3%)
*Years of experience in small ruminant breeding*	
3–10	6 (13.7%)
11–20	8 (18.2%)
21–30	8 (18.2%)
More than 30 years	22 (50.0%)

**Table 2:** Medicinal plants used for treatment of diseases in small ruminants (sheep and goats) in Maputo Province.

Scientific name	Family	Local name Xitsonga and Portuguese (P)	Voucher nr	Parts used	Preparation method, application route, and ailment treated	RFC	UV	District
*Acacia* sp	Fabaceae	—	DFE30/2019	L	Grind the leaves along with salt to treat wounds. Apply topically.	0.02	0.02	Mo
*Alantsilodendron pilosum* Villiers	Fabaceae	Tsenga	DFE31/2019	Fr	Char the material and then grind it into powder. Apply daily after cleaning the wound to treat ticks and wounds, including those on hooves.	0.05	0.05	Na
*Aloe marlothii* A. Berger	Asphodelaceae	Mangane and Nlhava	—	L	Slice and mix with water for diarrhea and listeriosis treatment. Grind when dry, add water to combat worms, and administer orally.	0.07	0.07	Bo and Na
*Aloe* sp	Asphodelaceae	Mangane	—	L	Grind or slice into pieces and mix with drinking water to treat any disease, discomfort, worms, or diarrhea. Grind and apply topically to treat wounds and ticks.	0.16	0.16	Mar, Bo, and Mo
*Aloe zebrina* Baker	Asphodelaceae	Mangane	DFE5/2018	L	Grind and mix with a single grain of potassium permanganate and water. Filter the mixture and administer it orally for treating worms and internal infections.	0.02	0.05	Mar
*Balanites maughamii* Sprague	Zygophyllaceae	Mudladlofo	DFE18/2018	L	Grind and apply topically to treat wounds. Mix with water and administer both orally and topically for treating worms.	0.05	0.05	Bo
*Cissus quadrangularis* L	Vitaceae	Txololuana, Manhalane, Txovane, and Tetenha	DFE11/2018	L, Ap, and sp	Grind the leaves, with or without water, and apply topically for wound and abscess treatment. To prevent licking, animal feces can be placed on top. In addition, consider adding salt, car lubricant oil, and alkaline battery residue to the ground leaves. Mix water with the leaves and either administer orally or use it to wash the skin for treating dermatitis, any disease, and weight loss. For treating bloody diarrhea and internal infections, mix the sap with 1 or 2 tablespoons of salt and washing powder. Grind the leaves and mix with drinking water to treat diarrhea and respiratory problems.	0.45	0.20	Bo, Mo, Na, and Mat
*Combretum molle* R. Br. ex G. Don	Combretaceae	Xiquitse	DFE34/2019	R	Boil the equivalent of one handful of the root in 2 liters of water until it reduces to 1.5 liters, then administer orally to treat diarrhea and respiratory problems.	0.02	0.05	Mat
*Crotalaria monteiroi* Trubert ex Baker f	Fabaceae	Licalahumba	DFE7/2018	R	Combine with water and administer orally for treating abdominal pain.	0.02	0.02	Bo
*Dietes iridioides* (L.) Sweet ex Klatt	Iridaceae	Xinalane and Baramachomane	DFE39/2019	Rh and L	Crush the rhizomes and/or leaves, mix with water (boiled or not), and administer orally to treat worms and diarrhea. Grind the leaves, moisten them with water, and apply inside the nose for treating cough.	0.07	0.07	Mat
*Dracaena* sp	Asparagaceae	Xiquendla	DFE50/2019	Wp	Grind, mix with a small amount of water, rub onto the skin, and then rinse to treat wounds.	0.02	0.02	Mat
*Elephantorrhiza elephantina* (Burch.) Skeels	Fabaceae	Xiurai	DFE25/2019	B from R	Cut a 40 cm piece of the bark into sections and add 2 liters of water. Leave it throughout the day and administer orally to treat worms.	0.02	0.02	Mo
*Euphorbia cuneata* Vahl	Euphorbiaceae	Espinhosa (P)	—	L	Grind along with *Aloe* sp. leaves and combine with drinking water for treating any disease.	0.02	0.02	Mar
*Euphorbia kirkii* (N.E.Br.) Bruyns	Euphorbiaceae	Mulheve, Thlovo, Ilholho, and Mahombole	DFE24/2019	Sp and B	Administer 1 or 2 drops of the sap near the skin around the eyes to treat keratoconjunctivitis and other eye diseases. Grind the stem bark and apply to the eyes for the treatment of keratoconjunctivitis.	0.20	0.02	Mo and Mat
*Euphorbia tirucalli* L	Euphorbiaceae	Nduthla	—	Sp and Ap	Administer 1 drop of the sap near the skin around the eyes for treating eye diseases. Grind the aerial part, mix it with water, and administer orally to manage diarrhea.	0.05	0.05	Mar and Bo
*Gymnosporia heterophylla* Loes	Celastraceae	Xilhangua	DFE33/2019	L	Char, grind, and add a cup of water and salt. Administer orally to treat paralysis, malaise, and worms. Secure the neck with a rope.	0.02	0.09	Mat
*Hyphaene coriacea* Gaertn	Arecaceae	M'hanga	—	Ap	Add to drinking water and administer orally to treat any disease.	0.02	0.02	Bo
*Indigofera tinctoria* L	Fabaceae	Mulhalantene	DFE32/2019	R	Burn and apply the resulting smoke to the eyes for the treatment of keratoconjunctivitis.	0.02	0.02	Na
*Momordica balsamina* L	Cucurbitaceae	Cacana	DFE44/2019	Ap	Grind a quantity of two handfuls of the aerial part, and then add 20 liters of drinking water to treat malaise and worms. Cut the plant, mix it with cold water until the color changes, and use the mixture to wash the animal for treating wounds and skin diseases.	0.05	0.09	Na and Mat
*Musa* sp	Musaceae	Bananeira (P)	—	L	Grind together with *Alantsilodendron pilosum* fruit and powder-dried *Nicotiana tabacum* leaves. Use the mixture to apply on wounds.	0.02	0.02	Na
*Nicotiana tabacum* L	Solanaceae	Tabaco (P)		L	Grind and apply to the wound (with or without worms), then secure with a tie.	0.05	0.02	Bo and Na
*Opuntia ficus-indica* (L.) Mill	Cactaceae	Xihaha	DFE40/2019	S	Grind a single stem, combine it with 2 liters of water, filter, and administer orally for treating diarrhea.	0.02	0.02	Mat
*Psydrax locuples* (K. Schum.) Bridson	Rubiaceae	Bandzane, Kipapetu, and Mucupampetu	DFE9/2018	L	Grind with or without water and apply topically to treat wounds (with or without worms) and furuncles.	0.07	0.05	Mar and Mo
*Scadoxus puniceus* (L.) Friis and Nordal	Amaryllidaceae	Ciquire	—	R	Peel, moisten with water, and apply to the wound.	0.02	0.02	Mat
*Securidaca longepedunculata* Fresen	Polygalaceae	Ntsatse	DFE8/2018	L	Administer orally for the treatment of any disease. Conversely, the animal self-consumes it when it has any disease.	0.02	0.02	Mar
*Sesamum senecioides* (Klotzsch) Byng and Christenh	Pedaliaceae	Lichéchua	DFE3/2018	Ap	Cut and administer orally for the treatment of worms, diarrhea, intestinal diseases, and multiple other conditions.	0.02	0.09	Mar
*Spirostachys africana* Sond	Euphorbiaceae	Xilangamalhu and Xilate	DFE28/2019	B from S, sp, and L	Dry the stem bark and grind it into powder; alternatively, grind the leaves, add the sap and water, and tie onto the wound.	0.05	0.02	Mo and Mat
*Strychnos* sp	Loganiaceae	Bandalhoko	DFE36/2019	L	Crush with or without water until a paste forms, and then apply it to the wound (on the skin or hooves). In addition, it is feasible to add a one-handed amount of alkaline battery powder.	0.05	0.02	Mat
*Strychnos spinosa* Lam	Loganiaceae	Massala	DFE16/2018	Fr, Sd, and R	Dry the pericarp and seeds, char, and apply the resulting ash to the wound. Alternatively, peel the root, combine it with water, and apply 2 drops in the ears for treating earache.	0.05	0.05	Bo
*Synaptolepis kirkii* Oliv	Thymelaeaceae	Vunguvane and Kama	DFE37/2019	R	Scrape with *Scadoxus puniceus* root, moisten it with water, and place it in the wound; or grind it along with *Strychnos* sp. leaves to create a paste. Apply an amount equivalent to one hand to the wound.	0.05	0.02	Mat
*Terminalia sericea* Burch. ex DC	Combretaceae	Conola	DFE6/2018	L and B from R	Cut the leaves or root bark, mix with water, and administer orally to treat abdominal pain, diarrhea, and cough	0.07	0.07	Bo, Mo, and Na
*Vernonia colorata* Drake	Asteraceae	Palhakufa,	DFE45/2019	L	Grind one or two handfuls of the leaves, along with *Terminalia sericea* leaves, and add 1 liter of water to treat diarrhea and cough. Grind with *Cissus quadrangularis* leaves and *Vernonia* sp. leaves, then apply to the wound.	0.05	0.07	Na
*Vernonia* sp	Asteraceae	Nhatelo	DFE47/2019	L	Grind together with *Cissus quadrangularis* leaves and *Vernonia colorata* leaves, and then apply it to the wound. Crush and rub over the entire body. Combine with drinking water and administer orally to treat wounds and for weight loss.	0.07	0.05	Na
*Zanthoxylum* sp	Rutaceae	Xinunguane	DFE49/2019	R	Combine 10 cm of the root with 4 *Vernonia* sp. leaves and *Cissus quadrangularis* aerial part. Apply onto the wound. In addition, incorporate Rapé (a local preparation based on *Nicotiana tabacum* leaves).	0.02	0.02	Na
Unidentified	—	Mamaruca	—	L	Grind, combine with 1 tablespoon of salt, and then apply it to the wound and secure it with a tie.	0.02	0.02	Mo
Unidentified	—	Nhalanova	—	S (Bulb)	Grind and rub onto the skin to treat scabies and mouth wounds.	0.02	0.05	Na
Unidentified	—	Pacama Xigomungomu	—	S	Grind, combine with water, filter, and administer orally for the treatment of diarrhea and worms.	0.02	0.05	Na
Unidentified	—	Bulele	—	L	Combine water, boil, and administer orally. Also, apply by rubbing onto the animal's skin to treat weight loss, malaise, edema, and skin blisters.	0.02	0.09	Na

L, leaves; R, root; Wp, whole plant; Fr, fruit; Sd, seeds; S, stem; B, bark; Sp, sap; Ap, aerial part; Rh, rhizome; Bo, Boane; Mar, Marracuene; Mat, Matutuíne; Na, Namaacha; Mo, Moamba.

**Table 3:** Fidelity level for the 3 most cited medicinal plants.

Plants	Ailment treated	FL
*Cissus quadrangularis* L	Wounds	1.00
	Diarrhea	0.14

*Euphorbia kirkii* (N.E.Br.) Bruyns	Keratoconjuntivitis	0.89

*Aloe* sp	Diarrhea	0.29
	Intestinal worms	0.29

**Table 4:** Analysis of factors associated with the use of plants for the treatment of small ruminants.

Parameters	Use of plants		Chi-squared		Fisher's exact
	No	Yes	*χ* ^2^	*P* value	*P* value
*Age*					
25–39	2	2	2,0341	0,7295	0,8415
40–59	6	12			
60–79	5	11			
80–89	0	3			
Did not remember	1	2			
*Gender*					
Female	2	5	0,0404	0,8406	1,0000
Male	12	25			
*Place of birth*					
Maputo	10	24	2,8577	0,5819	0,6537
Gaza	0	1			
Inhambane	3	2			
Nampula	0	1			
Not identified	1	2			
*Profession*					
Exclusively breeder	10	7	0,8774	0,3489	0,5087
Combining breeding with other profession	4	13			
*Religion*					
Christian	13	21	3,9152	0,2708	0,3498
Traditional African religion	0	4			
Muslim	1	2			
No religion	0	3			
*Residency*					
Marracuene	2	3	2,7557	0,7376	0,8038
Boane	1	5			
Moamba	2	8			
Namaacha	3	4			
Matutuine	6	9			
Katembe	0	1			
*Schooling*					
1^st^–5^th^ grade (former system)	8	8	4,0508	0,1319	0,1645
1^st^–12^th^ grade (actual system)	2	10			
Other (No schooling or literacy)	4	12			
*Years of experience in small ruminant breeding*					
3–10	3	3	3,7190	0,2934	0,3232
11–20	1	7			
21–30	4	4			
More than 30	6	16			

**Table 5:** Remedies other than plants used for the treatment of diseases in small ruminants (sheep and goats).

Identification	Common name	Preparation method, application route, and ailment treated	FC
Alkaline battery	—	Crush and grind into powder, then apply to the wound. In addition, consider using water, vaseline, or lubricant oil to adhere it to the skin.	6
Burned lubricant oil from car motor	—	Apply directly to the wound (it also prevents flies from entering the skin).	2
Water from car battery	—	Cleanse the wound.	1
Petroleum	—	Apply onto the wound.	1
Oil	—	Apply onto the wound.	1
Lichen	*Mulua*	Prepare a cold infusion using approximately 3 handfuls in 20 liters of water, along with a teaspoon of salt. Utilize this mixture to wash the animal, apply to the ears and nose, and administer orally for treating malaise and lack of appetite.	1
Lichen	*Ulele*	Boil in water, filter, and apply to the wound.	1
Internal shell of cuttlefish/cuttlebone (class Cephalopoda)	*Malelo*	Peel and grind into a powder, then blow into the eye using a reed (*Phragmites* sp. stem) to treat eye diseases.	1

FC, frequency of citation.

## Data Availability

The dataset used to support the findings of this study are available from the corresponding author upon request.

## Supplementary Materials

Supplementary Materials Supplementary file 1: IUCN status, endemicity, and nativity for identified plant species in the study in relation to Mozambique.
