# Supplementary material for: Ethnoveterinary Study of Plant-Based Remedies for Treating Diseases in Small Ruminants in Maputo Province, Mozambique
Source: Evid Based Complement Alternat Med. 2023 Oct 5;2023:1842870. doi: 10.1155/2023/1842870 (PMC10569895; doi:10.1155/2023/1842870)
Supplement: Supplementary Materials — Supplementary file 1: IUCN status, endemicity, and nativity for identified plant species in the study in relation to Mozambique. [file 1842870.f1.docx]

**Ethnoveterinary study of plant-based remedies for treating diseases in small ruminants in Maputo Province, Mozambique**

| IUCN status, endemicity, and nativity for identified plant species in the study in relation to Mozambique |
| --- |

| **Scientific name** | **IUCN status** | **Endemic to Mozambique?** | **Native or introduced?** |
| --- | --- | --- | --- |
| *Acacia* sp. | * | * | * |
| *Alantsilodendron pilosum* Villiers | Least concern | NE | Native |
| *Aloe marlothii* A.Berger | Least concern | NE | Native |
| *Aloe* sp. | * | * | * |
| *Aloe zebrina* Baker | Least concern | N/A | Native |
| *Balanites maughamii* Sprague | Least concern | N/A | Native |
| *Cissus quadrangularis* L. | Not available | N/A | Native |
| *Combretum molle* R.Br. ex G.Don | Least concern | N/A | Native |
| *Crotalaria monteiroi* Trubert ex Baker f. | N/A | N/A | Native |
| *Dietes iridioides* (L.) Sweet ex Klatt | N/A | N/A | Native |
| *Dracaena* sp. | * | * | * |
| *Elephantorrhiza elephantina* (Burch.) Skeels | N/A | N/A | Native |
| *Euphorbia cuneata* Vahl | Least concern | N/A | Native |
| *Euphorbia kirkii* (N.E.Br.) Bruyns | Least concern | N/A | Native |
| *Euphorbia tirucalli* L. | Least concern | N/A | Native |
| *Gymnosporia heterophylla* Loes. | Least concern | N/A | Native |
| *Hyphaene coriacea* Gaertn*.* | Least concern | N/A | Native |
| *Indigofera tinctoria* L. | N/A | N/A | Native |
| *Momordica balsamina* L. | N/A | N/A | Native |
| *Musa* sp. | * | * | * |
| *Nicotiana tabacum* L. | N/A | - | Introduced |
| *Opuntia ficus-indica* (L.) Mill. | Data deficient | N/A | Introduced |
| *Psydrax locuples* (K.Schum.) Bridson | Least concern | N/A | Native |
| *Scadoxus puniceus* (L.) Friis & Nordal | N/A | N/A | Native |
| *Securidaca longepedunculata* Fresen | Least concern | N/A | Native |
| *Sesamum senecioides* (Klotzsch) Byng & Christenh. | N/A | N/A | Native |
| *Spirostachys africana* Sond. | Least concern | N/A | Native |
| *Strychnos* sp. | * | * | * |
| *Strychnos spinosa* Lam. | N/A | N/A | Native |
| *Synaptolepis kirkii* Oliv. | N/A | N/A | Native |
| *Terminalia sericea* Burch. ex DC. | Least concern | N/A | Native |
| *Vernonia colorata* Drake | Least concern | N/A | Native |
| *Vernonia* sp. | * | * | * |
| *Zanthoxylum* sp. | * | * | * |
| Unidentified | * | * | * |
| Unidentified | * | * | * |
| Unidentified | * | * | * |
| Unidentified | * | * | * |

* It was not possible to determine, as the plant had not been described down to the species level

NE: Near-endemic - species were designated as those occur in five or fewer localities, besides Mozambique.

N/A: No information available
